# Antifungal Susceptibilities of *Cryptococcus neoformans*

**DOI:** 10.3201/eid1001.020779

**Published:** 2004-01

**Authors:** Lennox K. Archibald, Marion J. Tuohy, Deborah A. Wilson, Okey Nwanyanwu, Peter N. Kazembe, Somsit Tansuphasawadikul, Boonchuay Eampokalap, Achara Chaovavanich, L.Barth Reller, William R. Jarvis, Gerri S. Hall, Gary W. Procop

**Affiliations:** * Centers for Disease Control and Prevention, Atlanta, Georgia, USA; † Cleveland Clinic Foundation, Cleveland, Ohio, USA; ‡ Lilongwe Central Hospital, Lilongwe, Malawi; § Bamrasnaradura Hospital, Nonthaburi, Thailand; ¶ Duke University Medical Center, Durham, North Carolina, USA

**Keywords:** *Cryptococcus neoformans*, susceptibility testing, Malawi, Thailand, United States

## Abstract

Susceptibility profiles of medically important fungi in less-developed countries remain uncharacterized. We measured the MICs of amphotericin B, 5-flucytosine, fluconazole, itraconazole, and ketoconazole for *Cryptococcus neoformans* clinical isolates from Thailand, Malawi, and the United States and found no evidence of resistance or MIC profile differences among the countries.

Prompt identification of agents associated with emerging infectious diseases and documentation of resistance among these agents to available antimicrobial drugs depend on existing surveillance activities for emerging pathogens and antimicrobial resistance. Although the World Health Organization has undertaken initiatives (*1*) in these areas, surveillance of antimicrobial resistance in developing countries is lacking or has been generally ignored (*2*). Natural selective pressures exerted on microorganisms by routine, inappropriate, or excessive use of antimicrobial drugs are factors in the development of antimicrobial resistance. In tropical developing countries, unrestricted availability of antimicrobial drugs without prescriptions, suboptimal therapeutic regimens, blind empiric prescribing practices that are not epidemiologically directed, and lack of laboratory capacity or skilled personnel for susceptibility testing contribute to the spread of antimicrobial resistance (*2*). Although numerous studies have examined bacterial and mycobacterial resistance in the tropics, less is known about the susceptibility profiles of medically important fungi to antifungal agents (*3*–*5*). Given that only a few antimicrobial drugs may be available in developing countries because of limited resources or cost restrictions, the surveillance for resistance among common pathogens to available drug treatment is essential for appropriate patient care and improved patient outcome.

*Cryptococcus neoformans*, an opportunistic fungal pathogen that causes disease predominantly in immunocompromised patients, is a frequent cause of fatal mycotic infections among patients with AIDS (*6*). In sub-Saharan Africa, cryptococcal meningitis occurs in 30% of AIDS patients and is likely to remain a substantial cause of death in these patients unless highly active antiretroviral therapy becomes available (*6*–*8*). Until such a time, treatment with antifungal agents, including long-term, suppressive antifungal regimens, remains the only recourse.

## The Study

We sought to determine if substantial differences in susceptibility profiles to common antifungal agents existed among clinical isolates of *C. neoformans* from three geographically diverse areas. Sixty-five clinical isolates of *C. neoformans* from Malawi, Thailand, and the United States were available for study. The 16 isolates from Malawi and 29 isolates from Thailand were recovered from the bloodstream of febrile, adult inpatients during previous bloodstream infection studies in these regions (*9*,*10*). The 20 isolates from the United States were recovered from the bloodstream, lung tissue, cerebrospinal fluid, and other sterile sites in routine clinical practice in the clinical microbiology laboratories of the Cleveland Clinic Foundation and Duke University Medical Center. The yeast isolates from all of the countries were shipped to Duke University Medical Center for testing and maintained in frozen stock vials at –70°C. Sixty-five yeast isolates were recovered from the frozen stock vials on potato dextrose agar and incubated at 30°C for 48 hours. The antifungal susceptibilities of the isolates were determined by using the Sensititre YeastOne system (Trek Diagnostic Systems Ltd., West Sussex, England), which includes amphotericin B, 5-flucytosine, fluconazole, itraconazole, and ketoconazole. All isolates were incubated for 72 hours, according to the manufacturer’s instructions. Inoculum assessments were performed on all trays and were within acceptable limits. The trays were visually inspected, and the MICs were determined according to the manufacturer’s guidelines. Interpretive guidelines and breakpoints for susceptibility testing of *C. neoformans* are not yet available from the National Committee for Clinical Laboratory Standards (NCCLS); therefore, only MIC comparisons were performed (*11*).

For isolates from each country, we recorded the MIC at which 50% of the isolates were inhibited (MIC_50_) and the MIC at which 90% of the isolates were inhibited (MIC_90_) and determined the MIC geometric mean for each therapeutic agent. We compared the MIC geometric means for the three countries with a one-way analysis of variance (ANOVA) to determine if significant differences existed. Additional comparisons between the MIC_50_ and MIC_90_ were not undertaken, since these were within one dilution of one another.

The *C. neoformans* isolates from the United States, Thailand, and Malawi demonstrated similar susceptibility profiles to the common antifungal agents against which they were tested (Table 1). The percentage of isolates inhibited at each concentration of antifungal agent over the full dilution series is summarized in Table 2. The isolates from the three countries did not differ significantly in their susceptibility to fluconazole (p = 0.198), itraconazole (p = 0.163), 5-flucytosine (p = 0.713), or ketoconazole (p = 0.531). The geometric mean of the MIC values for amphotericin B in Thailand, the United States, and Malawi was 1.2 μg/mL, 1.4 μg/mL, and 1.6 μg/mL, respectively. These mean values were significantly (p = 0.019) different.

**Table 1:** *Cryptococcus neoformans* susceptibility results

Antifungal agent	MIC range (μg/mL)	MIC_50_ (μg/mL)	MIC_90_ (μg/mL)	MIC geometric mean (μg/mL)
U.S. isolates (N = 20)				
Amphotericin B	1–2	1	2	1.4
Fluconazole	1–16	8	8	5.1
Itraconazole	0.016–0.125	0.06	0.125	0.06
5-Flucytosine	2–8	4	8	5.1
Ketoconazole	<0.008–0.250	0.06	0.06	0.05
Thailand isolates (N = 29)				
Amphotericin B	0.5–2	1	2	1.2
Fluconazole	4–160	8	16	7.7
Itraconazole	0.030–0.125	0.06	0.06	0.06
5-Flucytosine	2–8	4	8	4.6
Ketoconazole	0.030–0.250	0.06	0.125	0.07
Malawi isolates (N = 16)				
Amphotericin B	1–2	2	2	1.6
Fluconazole	4–32	8	16	7.6
Itraconazole	0.030–0.125	0.03	0.125	0.05
5-Flucytosine	1–16	4	8	4.5
Ketoconazole	0.016–0.250	0.03	0.25	0.03

**Table 2:** Percentage of *Cryptococcus neoformans* isolates susceptible at each MIC dilution

	% Susceptible		
MICs (μg/mL)	U.S. isolates	Thailand isolates	Malawi isolates
Amphotericin B			
0.5		3	
1	50	72	31
2	100	100	100
Fluconazole			
1	5		
2	30		
4	40	21	25
8	90	83	87
16	100	100	94
32			100
Itraconazole			
0.016	5		
0.030	15	14	50
0.060	75	93	87
0.125	10	100	100
5 Flucytosine			
1			6
2	5	7	12
4	60	72	69
8	100	100	94
16			100
Ketoconazole			
<0.008	5		
0.016	20		12
0.030	35	14	62
0.060	90	79	75
0.125	95	93	81
0.250	100	100	100

## Conclusions

Resistance to antifungal drugs is rare among clinical isolates of *C. neoformans* but has been reported (*4*,*12*). The use of antifungal agents, particularly in long-term suppressive regimens, has raised concern about the development of drug resistance in *C. neoformans*. However, an extensive survey of the susceptibility profiles of clinical isolates of *C. neoformans* at a university hospital during 1987 to 1994 helped to allay these fears by indicating no emergence of resistance (*13*).

This study also demonstrates no evidence of resistance among clinical isolates of *C. neoformans* from Thailand, Malawi, and the United States. For each country, the MIC_50_ and MIC_90_ of isolates to commonly used antifungal agents were within one dilution from each other. In addition, the MIC ranges were similar. Statistical comparison of the MIC geometric means confirmed that no significant differences existed between the three regions for fluconazole, itraconazole, 5-flucytosine, or ketoconazole. The only statistically significant differences were observed for amphotericin B susceptibilities; however, this difference was believed to be clinically irrelevant since the MIC geometric means for amphotericin B were 1 - 2 μg/mL, or within one dilution. Our documentation of the absence of resistance among *C. neoformans* isolates from the United States is consistent with data published by the Centers for Disease Control and Prevention, which showed in vitro resistance to antifungal agents to be uncommon and unchanged among *C. neoformans* isolates from 1992 to 1998 (*14*).

 The similarity between the MICs of *C. neoformans* isolates from Malawi and the United States concurs with data from a previous study of 164 African and 402 North American clinical isolates of *C. neoformans* isolates that were tested and found to be susceptible to fluconazole and other triazoles, with over 99% inhibited by concentrations of fluconazole <32 μg/mL (*5*). The MIC_50_ and MIC_90_ in that study were lower than those in this study, although the YeastOne trays have been found to agree well with the NCCLS reference method for itraconazole and the other azoles (*15*). Also, the MICs of fluconazole documented in our study are similar to those previously reported for isolates of *C. neoformans* from the United Kingdom and Uganda (*3*,*4*); the MICs of 5-flucytosine in our study also were similar to those previously reported for *C. neoformans* isolates from Uganda (*4*). The itraconazole MICs documented in our study were lower than those reported for isolates from the United Kingdom, Africa, and the United States (*4*,*5*). The differences between the susceptibility profiles of *C. neoformans* to itraconazole reported in our study and those reported previously may be due in part to the poor solubility of this antimicrobial agent in an aqueous solution.

Using a standardized testing method, we found no significant or clinically meaningful differences between the antifungal susceptibility profiles of clinical isolates of *C. neoformans* from the United States, Thailand, and Malawi. Although rare strains of *C. neoformans* with elevated MICs to some antifungal agents may exist, they were not detected in this sampling of clinically significant *C. neoformans* isolates and, therefore, do not appear to be prominent in Cleveland, Ohio; Durham, North Carolina; Bangkok, Thailand; or Lilongwe, Malawi.
